# The histone gene family in *Drosophila simulans* carries consistent species-specific nucleotide polymorphisms distinct from that of *Drosophila melanogaster*

**DOI:** 10.17912/micropub.biology.002085

**Published:** 2026-05-15

**Authors:** Sierra Falcone, Annalise Weber, Salaam Awad, Leila Rieder

**Affiliations:** 1 Biology Department, Emory University, Atlanta, GA, US

## Abstract

*Drosophila melanogaster* is a powerful model system to study histone gene regulation. However, *D. melanogaster* histone coding sequences are nearly identical, constraining use of this model in elucidating the regulation of individual members of this gene family. While we found similar constraints in *D. simulans,* we confirmed species-specific SNPs between *D. simulans* and *D. melanogaster* histone coding sequences and discovered these SNPs are consistent across *D. simulans* histone gene paralogs. We suggest that the SNPs between species–and the ability for *D. melanogaster* and *D.* *simulans* to hybridize–open new avenues to explore evolutionary conservation of histone gene regulation between similar species.

**Figure 1. Drosophila simulans has a similar histone locus composition as Drosophila melanogaster, but species-specific single nucleotide variation:** **A.** The composition of the *D. simulans* histone genes based on long read and Sanger sequencing. Annotation of a long-read assembled *D. simulans* genome suggested the *D. simulans* histone genes are arranged in 15 quintet arrays. The original *D. simulans* cloned H3 sequence from previous work (Tsunemoto and Matsuo 2001; Matsuo 2000; green box) was confirmed by the long-read assembled genome (Kim et al. 2021). Long-read sequencing reveals microheterogeneity between the H3 genes in arrays (pink highlight). However, Sanger sequencing of the H3 coding sequence shows homogeneity between the H3 genes. Both *Drosophila* species have unique single nucleotide polymorphisms in the H3 gene (purple highlight), determined through PCR and sanger sequencing. Sequences are representative H3 sequences from the 20 sanger sequenced colonies from a single fly. **B.** Immunofluorescence staining of *Drosophila* polytene chromosomes. Multi-sex combs (Mxc; magenta) localizes specifically to the histone locus. *D. simulans* and *D. melanogaster* both carry a single histone locus. **C.** The copy number of *D. simulans* (pink) and *D. melanogaster* (blue) H3 genes is similar, as assayed using genomic DNA droplet digital PCR and compared to the diploid gene *gapdh* . The error bars represent a 95% confidence interval of this measurement.

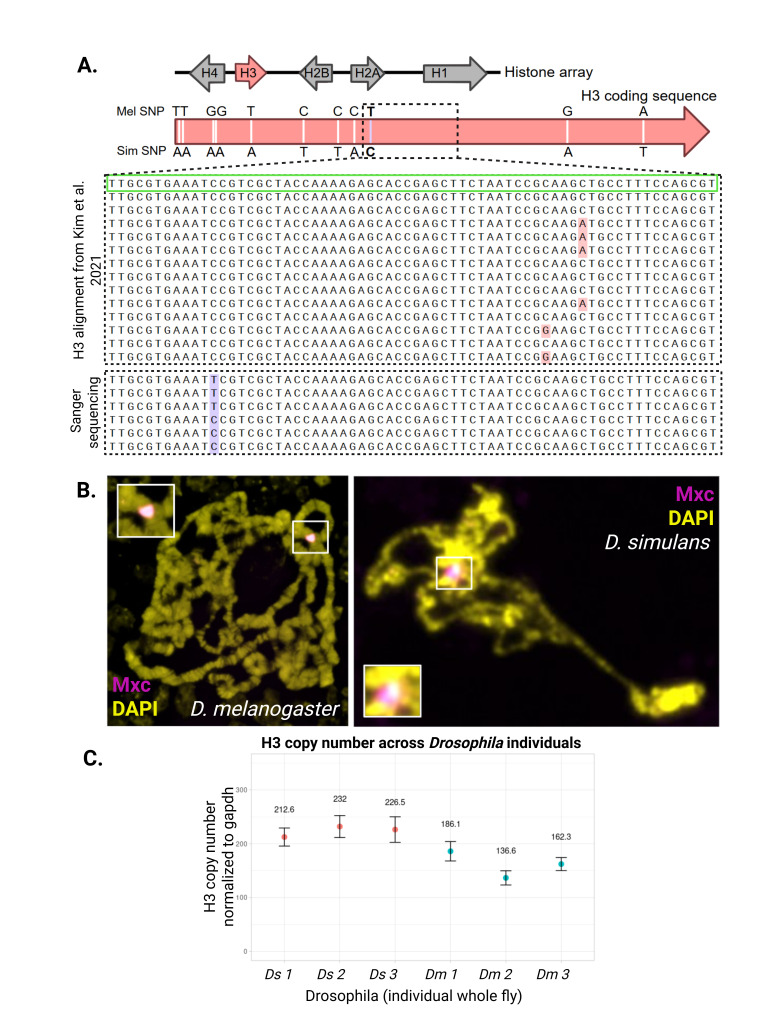



## Description

Histone proteins are responsible for compacting and organizing genomic DNA. These proteins are encoded by a multigene family in many eukaryotic genomes (Eirín-López et al. 2009; Maxson et al. 1983; Chabouté et al. 1993) . *Drosophila melanogaster,* whose genome hosts a single histone locus on chromosome 2L composed of ~100 tandemly repeated histone gene arrays (Bongartz and Schloissnig 2019; Shukla et al. 2025; McKay et al. 2015) , has emerged as a powerful model with which to study histone gene regulation. This organism is particularly useful because a precise deletion of the endogenous histone locus (Crain et al. 2024; Günesdogan et al. 2010) can be rescued using large histone array transgenes (Crain et al. 2024; Meers et al. 2018; Günesdogan et al. 2010; McKay et al. 2015) . However, these tools are not yet able to address a longstanding question about the regulation of repetitive, similar genes: does the cell titrate expression from all genes equally, or are only some members of the gene family expressed?

These questions have historically been impossible to address in *D. melanogaster* due to extreme homogeneity between histone coding sequences within the fly genome, driven by concerted evolution (Arnheim 1983; Smith 1974; Hodkinson and Rieder 2024; Crain et al. 2024) . It is impossible, for example, to map RNA-sequencing data back to the histone gene of origin. Thus, we sought to identify a new species in which to study the expression of individual members of the repetitive, clustered histone gene family. We investigated the histone gene sequences in the genomes of the *melanogaster* subgroup, with the goal of identifying natural sequence variation between histone coding sequences within a single genome.

While genome assemblies exist for many *Drosophila* species, they are often assembled through the use of short-read sequencing techniques, which do not map properly to the repetitive histone locus. For example, only 23 histone gene arrays are annotated in the well-used dm6 ( *D. melanogaster* ) genome, despite evidence of ~100 arrays (Bongartz and Schloissnig 2019; Shukla et al. 2025; McKay et al. 2015; Lifton et al. 1978) . We reasoned that recent genome assemblies of 93 Drosophilid species, obtained through long-read sequencing, provided a new avenue to search for an alternative model system (Kim et al. 2021) . We annotated histone genes in the long-read assembled genome of *Drosophila simulans* , as this species diverged from *D. melanogaster* only 2.5 million years ago *.*

In the *D. simulans* assembled genome from Kim et al, we found only 15 histone arrays at a single locus, a stark contrast to the ~100 clustered histone arrays in *D. melanogaster* (Bongartz and Schloissnig 2019; Shukla et al. 2025; McKay et al. 2015) . Previous work cloned and sequenced a single histone array in *D. simulans* and we found the same reported H3 coding sequence (Matsuo 2000) ( Fig. 1A, green box). However, this work did not capture the potential heterogeneity between histone genes in different arrays. By comparing H3 coding sequences from all 15 arrays, we were excited to find SNVs between them ( Fig. 1A, pink highlight). *D. simulans* therefore seemed like a promising new system with which to investigate the expression patterns of subsets of histone genes within a locus.

To confirm that *D. simulans* carries a single histone locus (Rieder et al. 2017) , we performed polytene chromosome immunostaining for Multi-sex combs (Mxc), a conserved protein that only localizes to the histone genes (White et al. 2011) . As in *D. melanogaster* , Mxc targets a single locus near the chromocenter in *D. simulans* , indicating a single, large histone locus ( Fig. 1B ). To confirm histone gene copy number in *D. simulans* , we performed droplet digital PCR (ddPCR), a method similar to qPCR that determines the exact copy number of a target. We designed primers and TaqMan probes that are universal to both the *D. melanogaster* and *D. simulans* H3 coding sequences, as well as to the diploid control gene *gapdh* . We performed ddPCR on genomic DNA from both species and found that the H3 copy number in *D. simulans* is similar to that of *D. melanogaster* ( Fig. 1C ), discordant with our initial observations from the long read assembled *D. simulans* genome (Kim et al. 2021) . The numbers of H3 genes in individual flies varied slightly in both species, consistent with documented variation in the number of histone arrays between populations of *D. melanogaster* (Shukla et al. 2025) . As all H3 genes are members of quintet histone gene arrays ( Fig. 1A ) in *D. melanogaster* (Lifton et al. 1978) , our observations suggest that the genomes of the closely related *D. simulans* and *D. melanogaster* carry a similar number of histone gene arrays. Between the discrepancies in copy number that we observed, and the high error rate of long-read sequencing technologies, there may be limits in predicting accurate copy number of genes from repetitive regions. However, two groups have successfully assembled only the *D. melanogaster* histone locus using long read sequencing (Bongartz and Schloissnig 2019; Shukla et al. 2025) suggesting the *method* of genome assembly is important to accurately assemble highly repetitive regions.

Since long-read sequencing is prone to artifacts (Weirather et al. 2017) , we next sought to confirm sequence heterogeneity within the H3 coding sequences in *D. simulans* ( Fig. 1A ). We performed PCR on *D. simulans* genomic DNA from five separate adult flies, targeting the entire H3 coding sequence (411bp). We cloned the PCR products and performed Sanger sequencing on ~20 individual colonies from each fly. Surprisingly, we observed near complete sequence homogeneity between *D. simulans* H3 coding sequences both within individual genomes and between individuals ( Fig. 1A ; Supplemental Figure 1). Our Sanger sequencing contradicts the heterogeneity we observed in the long-read assembled genome ( Fig. 1A ). This discrepancy is likely due to the high error rate of long-read sequencing techniques (Weirather et al. 2017) , and indicates that genome-wide long-read assemblies should not be used to investigate sequence heterogeneity in repetitive regions. However, when we previously compared histone array sequences in *D. melanogaster* using long-read assemblies focused on the repetitive histone locus (Bongartz and Schloissnig 2019; Shukla et al. 2025) , we found little sequence variation (Hodkinson and Rieder 2024) .

While sequence homogeneity between *D. simulans* histone genes indicated that we could not investigate expression patterns of individual histone genes in this species, we revealed 11 distinct, species-specific, single nucleotide polymorphisms (SNPs) *between* the H3 coding sequences of *D. simulans* and *D. melanogaster* ( Fig. 1A, Fig. S1). This observation is congruent with prior work showing the existence of species-specific SNPs in the histone coding sequences between other species in the *melanogaster* subgroup (Tsunemoto and Matsuo 2001; Kakita et al. 2003; Matsuo 2000) . This prior investigation also assumed single histone arrays were representative of all histone coding sequences within a species. Here, we confirm species-specific SNPs are consistent across all *D. simulans* histone genes. The accumulation of synonymous mutations across a locus is consistent with concerted evolution: a single gene acquires a mutation that is then duplicated through unequal crossing over or biased gene conversion (Elder and Turner 1995; Smith 1974; Arnheim 1983) . The synonymous mutation eventually becomes homogenized between paralogs at a single locus within a species.

The species-specific SNPs, which are homogenous within each species but different between species ( Fig. 1A ), present a unique opportunity for future avenues of research since *D. simulans* and *D. melanogaster* can hybridize (Sturtevant 1920) . Therefore, histone transcripts from *D. simulans* - *D. melanogaster* hybrids can be differentiated using short read techniques such as RNA-sequencing. In addition, genetic tools normally constrained to *D. melanogaster* , including GAL4-driven RNAi, mutant alleles, and existing histone array transgenes (McKay et al. 2015; Crain et al. 2024; A et al. 2023; Kurihara et al. 2020; Rieder et al. 2017) , can also be strategically used in hybrids. We suggest that the ability to differentiate transcripts from parental histone loci in hybrids presents a new opportunity to explore mechanisms of histone gene regulation.

## Methods

**Annotation of long read sequencing data**

We obtained *D. simulans* long read sequencing data from NCBI SRA and GenBank under NCBI BioProject PRJNA675888. We imported the *D. simulans* genome into SnapGene software (version 8.1.1) for all annotations. We hand-annotated histone gene sequences using the protein sequence search tool in SnapGene. We aligned histone gene sequences using the SnapGene MUxzSCLE alignment tool (Edgar 2004) .

**Polytene immunofluorescence microscopy**

We performed polytene squashes from *Drosophila melanogaster* ( *y,w* ) and *Drosophila simulans* (wild type; from the Cornell Species Stock Center, #SGA34) third instar larvae. We fixed salivary gland tissue by incubating tissue in Fix 1 (4% formaldehyde, 1% Triton X-100, in 1xPBS) for 1 min, Fix 2 (4% formaldehyde, 50% glacial acetic acid) for 2 min and Fix 3 (1:2:3 ratio of lactic acid:water:glacial acetic acid) for 5 min. After fixing, we squashed salivary glands onto slides using mechanical disruption of the slide cover. We then dipped slides into liquid nitrogen for a few seconds and flicked off the cover slips. We placed slides in 90% EtOH at -20℃ for storage. We washed slides in 1xPBS for 15 min at RT with gentle agitation and then 1% TritonX-100 in 1xPBS for 10 min. We blocked slides with 0.5% BSA in 1xPBS for one hour. We incubated slides with diluted guinea pig anti-Mxc primary antibody (1:5,000) (White et al *.* 2011, gift from the Duronio and Marzluff labs) with 0.5% BSA in 1xPBS overnight in a humid chamber at 4℃. We then washed slides 3X5 min in 1xPBS. We incubated samples with AlexaFluor secondary 647 antibodies (Thermo Fisher Scientific, A21450) at a concentration of 1:1,000 for two hours in a covered humid box. We washed slides 3x5 min in 1xPBS and then incubated them in DAPI (1:1000 in 1xPBS) for 10 min. We washed slides for 5 min in 1xPBS and mounted samples using ProLong Antifade Diamond (Invitrogen P36965). We imaged slides on A Zeiss AXIO Lab A1 scope with Zen Blue software and false colored images using FIJI imaging software (Schindelin et al. 2012) .

**Genomic DNA extraction**

We placed *D. simulans* (5 biological replicates) and *D. melanogaster* (1 biological replicate) adult flies in 1.5mL microcentrifuge tubes and euthanized flies at -80℃ for 10 min. We thawed the flies, and added 50uL of squish buffer (10 mM Tris-Cl pH 8.0, 1 mM EDTA, 25 mM NaCl and 200 ug/ml fresh Proteinase K). We used a P200 pipette tip to "squish" the fly and mechanically agitate the solution and used an electric pestle to finish dissociating tissue. We incubated the mixture at 37℃ for 30 min and then 95℃ for two min to deactivate Proteinase K. We centrifuged the solution at 14,000 rpm for 7 min and aliquoted 30uL of supernatant to store at -20℃.

**Droplet digital PCR**

We designed primers and TaqMan probe sequences to be universal to *Drosophila melanogaster* and *Drosophila simulans* Histone 3 (H3) and *gapdh* exon sequences (see Reagents section). We diluted primers, probes and water together in a 9:25:32 ratio, respectively, to form a 20x master mix. We incorporated the primer/probe master mix and 0.02u/L per sample of restriction enzyme BamH1-HF (New England Biolabs R3136S) into the ddPCR master mix (BioRad 1863023). We selected Bam-H1 because it is predicted to cut once in every histone array in both species. We diluted the genomic DNA of both species to ~0.2ng/ul due to the high copy number of histone genes in the genomes. We performed droplet generation and transferred droplets to PCR plates according to the Bio-Rad QX200 standard protocol (Instruction Manual, QX200 Droplet Generator – Bio-Rad).

We performed the PCR reaction in a thermocycler as follows: we performed the enzyme activation step at 95℃ for 10 min, followed by 40 cycles of two-step cycling (denaturing at 94℃ for 30 sec, annealing at 63℃ for 1 min), and a final melting step of 98℃ for 10 min and a 4℃ hold.

We transferred the PCR droplets into the Bio-Rad QX200 droplet reader system (Bio-Rad, 1864001). We performed data acquisition and droplet reading according to the Bio-Rad QX200 standard protocol (Instruction Manual, QX200 Droplet Generator – Bio-Rad) and obtained the absolute quantity of the copy number using BioRad QuantaSoft software. We exported and visualized the data using RStudio (Posit team (2025). RStudio: Integrated Development Environment for R. Posit Software, PBC, Boston, MA. URL http://www.posit.co/.). We performed digital droplet PCR under advisement by, and using instruments from, the Emory Genomics Core.

QX Manager v2.2 Standard calculated the copy number of *D. simulans* and *D. melanogaster* H3 genes using a reference gene, usually diploid. Copy number = ([H3]/[gapdh]) * 2

**Cloning and Sanger sequencing of H3 coding sequences**

Using genomic DNA from 5 adult flies (3 females, 2 males), we performed PCR amplification of the entire H3 gene coding sequence using Q5 Taq polymerase in *Drosophila melanogaster* and *Drosophila simulans* . Primers for both species are found in the Reagents section. We then ligated PCR amplicons into a BS II KS vector plasmid (obtained from O. Laur, Emory Integrated Genomics Core) and transfected them into Dalpha5 cells. We plated cells on Carbenicillin LB plates. We miniprepped 20 Colonies per species using a QIAprep Spin Miniprep Kit (Qiagen Cat. No. #27104). We sent samples to Plasmidsaurus for sequencing. We aligned sequences using SnapGene MUSCLE alignment tool (Fig. S1).

## Reagents

Primer sets and TaqMan probes for ddPCR:

H3 Forward primer 5'-AGCGTCTGGTGCGTGAAATC-3'

H3 Reverse primer 5'-ATGAATGGCACACAAGTTGG-3'

H3 TaqMan probe sequence 5'-TGCGATTCCAGAGCTCGGCGGTTAT-3' (FAM/zen)

GAPDH Forward primer 5'-GACGAAATCAAGGCTAAGGTCG-3'

GAPDH Reverse primer 5'-AATGGGTGTCGCTGAAGAAGTC-3'

GAPDH TaqMan probe 5'-TCCTCATCGGTGTAGCCCAGGATTCCCTT-3' (HEX/Zen)

Primer sets for H3 gene cloning and Sanger Sequencing:

Forward primer 5'-GCTCGTACCAAGCAAACTGC-3'

Reverse primer 5'-GAATGCGTCGCGCTAACTGGA-3'
